# Usefulness of the Blink Reflex in Diagnosing Isolated Infraorbital Neuropathy following Midface Augmentation with AlloPlastic Facial Implants: A Case Report

**DOI:** 10.3390/life12081122

**Published:** 2022-07-26

**Authors:** Byoung Hoon Kim, Haseon Yang, Myung Chul Yoo

**Affiliations:** 1 Department of Physical Medicine & Rehabilitation, College of Medicine, Kyung Hee University, Seoul 02447, Korea; 25497@khmc.or.kr (B.H.K.); didskago@khmc.or.kr (H.Y.); 2 Department of Medicine, Graduate School, Kyung Hee University, Seoul 02447, Korea

**Keywords:** infraorbital nerve injury, facial allograft implant, blink reflex study, case report

## Abstract

For the preoperative evaluation of infraorbital nerve injury, most clinicians depend on the patient’s subjective symptoms or judgements, lacking a generalized and objective evaluation method. Due to the limitations in subjective evaluations for accurate diagnosis of infraorbital nerve injury, we used the blink reflex to objectively evaluate injury to the infraorbital nerve. A 49-year-old female, who had previously undergone midface augmentation with alloplastic implants, presented with sensory loss in the left upper lip, nose tip, and lower palatal area. Physical examination revealed sensation loss in the area innervated by the infraorbital nerve. Facial three-dimensional computed tomography did not identify compression of the infraorbital nerve. The blink reflex study of the infraorbital nerve was evaluated preoperatively. After the patient was diagnosed with injury along the infraorbital nerve pathway from alloplastic facial implants, she underwent facial implant removal with decompression surgery. The patient experienced a significant decrease in hypoesthesia, and her sensory function improved. The blink reflex study was an effective method to objectively diagnose infraorbital neuropathy. Therefore, clinical use of the blink reflex study as an electrophysiological diagnostic tool is recommended to investigate infraorbital nerve injuries.

## 1. Introduction

Patients with maxillary lesions present with various symptoms, some of which are caused by trigeminal nerve deficits, such as loss of temperature sensation or tactile deficits. The infraorbital nerve (ION), a branch of the maxillary nerve and second division of the trigeminal nerve, supplies sensation to the middle parts of the face. Patients with ION injury often experience discomfort, such as stinging pain, hypoesthesia, or dysesthesia. Significant symptoms are numbness of the upper lip, lateral nose, cheek, and midface of the affected side [1].

In clinical practice, ION damage due to trauma is not uncommon, and in many cases, the ION injury primarily results from midface trauma, which is mainly associated with orbitozygomatic complex fractures, often leading to sensory disturbances. Clinicians typically examine ION function abnormalities based on patient symptoms and a physical examination as a standard nerve conduction study is not possible anatomically. Therefore, clinicians usually make decisions for surgical treatment based on patients’ subjective symptoms, such as dysesthesia or stinging pain, due to lack of objective methods to evaluate the ION. Although it is classically taught that ION injury accompanies such symptoms, it is not clear to what extent damage to the ION can be detected preoperatively and to what extent this persistent damage is clinically significant. In addition, if an ION injury is suspected, especially if the lesion is not observed by paranasal or three-dimensional facial CT (computed tomography), it is more difficult for clinicians to determine whether surgery is needed, emphasizing the need for development of objective evaluation tools.

Based on this background, the blink reflex study can be used as an objective and non-invasive method to test for nerve injury. In general, the blink reflex study is useful in detecting abnormalities of the trigeminal and facial nerves. Furthermore, it has been used to detect abnormalities in the central nervous system, including the pons and medullar areas, as well as the trigeminal and facial nerves. Moreover, eye blinks have proven useful in a wide range of applications, with studies showing that an increased eye blink rate is associated with an increase in cognitive load [2], that eye blinks can serve as both a source of information for detecting EEG-based driver drowsiness, as well as a potential source of artifacts [3], and that blink rate variability is a promising indicator of mental state [4].

The decision for surgical performance by clinicians is important, and preoperative diagnosis of ION injury is essential for recovery and prognosis. To the best of our knowledge, few studies have been conducted on the evaluation of ION deficits using a blink reflex study [5,6]. In particular, there has been no case report of an isolated ION injury due to midface augmentation with alloplastic facial implants, as in the present case. We aimed to utilize the blink reflex study to illustrate a rare finding of ION injury and to report isolated infraorbital neuropathy as a surgical complication after midface augmentation with alloplastic implants.

## 2. Case Presentation

A 49-year-old female visited the outpatient clinic of the Department of Plastic Surgery at the University Hospital, due to sensory loss in her left upper lip, nose tip, and lower palatal area. Her symptoms began three months prior to presentation. She had a history of multiple plastic surgeries, including local blepharoplasty 15 years prior, facelift with revision blepharoplasty in 2014, and paranasal augmentation with lower lip augmentation in 2015.

**Figure 1:**
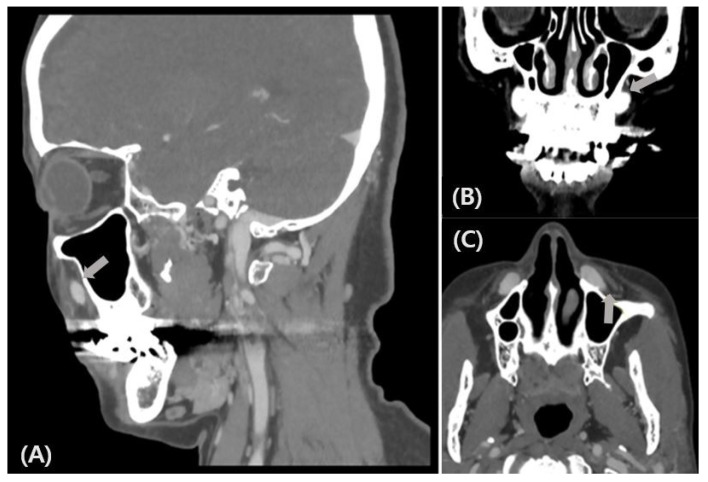
Sagittal (**A**), coronal (**B**) and axial (**C**) facial computed tomography images in a 49-year-old woman show that the left infraorbital nerve is intact around the infraorbital foramen and travels to the peripheral side of the foreign body. A prominent nerve contact or compression is not apparent, but the contact of the peripheral branch of the left infraorbital nerve (arrows in **A**–**C**) is possible.

The patient was referred to the Department of Physical Medicine and Rehabilitation for an electrodiagnostic study to determine the extent of facial nerve injury. There was no tenderness or swelling of the left nasolabial groove area, no facial palsy, and her facial function, which was assessed using the House–Brackmann grading system, was grade I. Facial examination indicated a sensory deficit in the left side of the lateral nose, upper lip and anterior cheek. An ION branch injury in the trigeminal nerve was clinically suspected as the cause of the patient’s symptoms. Facial needle electromyography was carefully performed to avoid the frontalis muscle due to the foreign implant in her forehead. A facial nerve conduction study and blink reflex study were performed in the supraorbital nerve, with normal findings. A three-dimensional facial CT was performed to confirm ION compression due to the foreign body implant. Facial CT findings revealed triangular-shaped areas of high density on the anterior sides of both maxillary sinuses, which were judged as foreign bodies (Figure 1). The left ION was intact around the infraorbital foramen and traveled to the peripheral side of the foreign body. Prominent nerve contact or compression of the ION was not observed; and it was not possible to confirm whether the peripheral branch of the left ION had been damaged by the implant.

**Figure 2:**
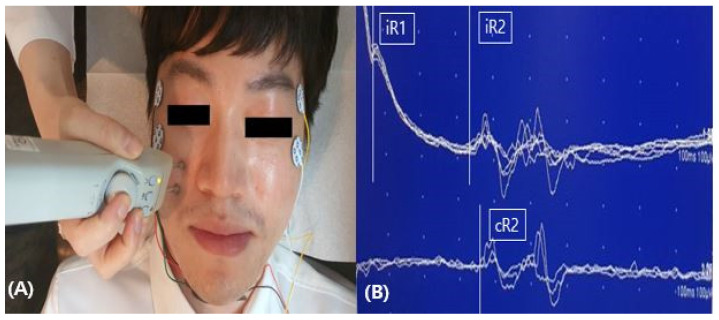
Evaluation of the blink reflex study. (**A**) Stimulation of infraorbital nerve: reference electrode on bilateral temples, ground electrode on chin, with stimulation above infraorbital foramen, active electrodes on bilateral lower eyelids. (**B**) Waveform of the infraorbital blink reflex of the normal patient.

**Figure 3:**
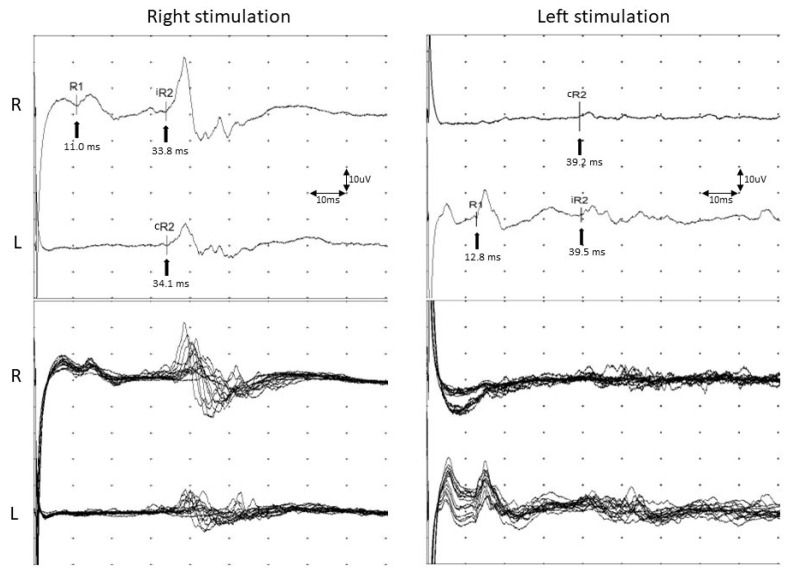
Evaluation of the blink reflex after infraorbital nerve stimulation before surgery to remove the implant. Following stimulation of the left side, the left ipsilateral early wave (R1) and ipsilateral late wave (R2) were more delayed than right ipsilateral R1 and R2 waves. Arrows indicate the latency of each response. Abbreviations: iR2, ipsilateral R2; cR2, contralateral R2.

**Figure 4:**
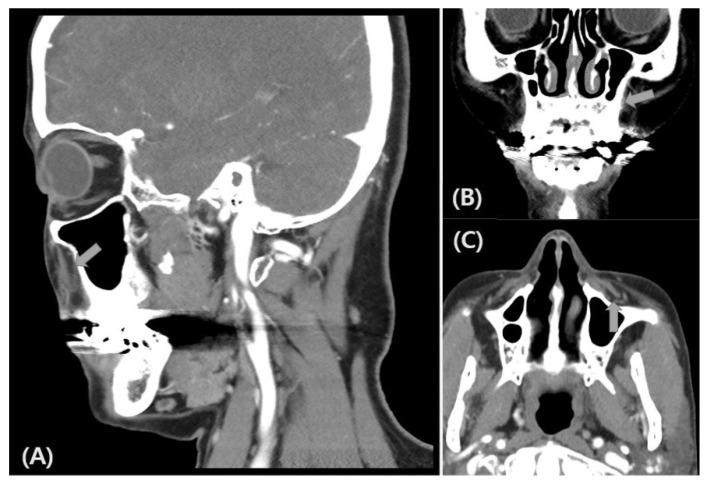
Sagittal (**A**), coronal (**B**) and axial (**C**) facial computed tomography images after implant removal. There is no implant at the nearby infraorbital nerve (arrows in **A**–**C**).

**Figure 5:**
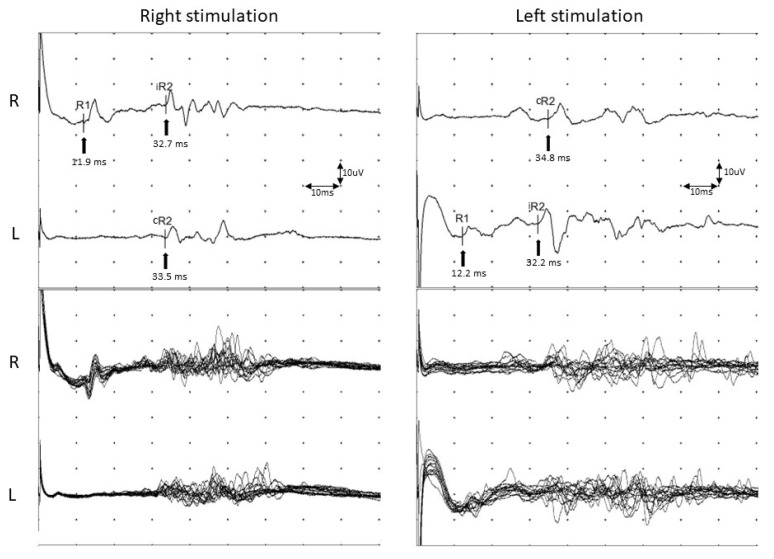
Evaluation of the postoperative blink reflex after infraorbital nerve stimulation. The left ipsilateral R2 wave was much shorter than that observed prior to implant removal. Arrows indicate the latency of each response. Abbreviations: iR2, ipsilateral R2; cR2, contralateral R2.

To evaluate dysfunction of the ION, we applied the same method from Ohki’s study to stimulate the ION [5]. The patient was asked to gently close her eyes in the supine position, and active recording electrodes were attached to the orbicularis oculi muscle at the bilateral lower eyelids. Reference electrodes were attached to the bilateral temples, lateral to the eyes. Electrical stimulation was applied to the skin over the infraorbital foramen. The ground electrode was attached to either the chin or forehead (Figure 2A). The stimulation intensity was 20–25 mA, with a duration of 0.1 ms at a frequency of 0.5 Hz. Sierra (Cadwell, GA, USA) was used with the following settings: sensitivity of 200 uV/division, sweep speed of 10 msec/division, and filter at 100–1500 Hz (Figure 2B). In the blink reflex study of the ION when the left side was stimulated, the left ipsilateral early wave (R1) and late wave (R2) were delayed compared to the right ipsilateral R1 and R2. The blink reflex was normal when the right side was stimulated, indicating a deficit in the afferent pathway of the left ION (Table 1, Figure 3). Based on these results, the plastic surgeon decided to perform implant removal and decompression surgery to treat the patient’s left infraorbital neuropathy. By the one-month follow-up visit, the patient had experienced a significant decrease in hypoesthesia, resulting in improved sensory function. No postoperative complications were experienced. The follow-up facial CT findings showed that the foreign body in the maxillary area had been removed (Figure 4). In addition, the left ipsilateral R1 was not significantly different from the previous results, but the left ipsilateral R2 was much faster than before implant removal (Table 1, Figure 5). Written informed consent was obtained from the patient for publication of this case report and the accompanying images.

## 3. Discussion

For the preoperative evaluation of infraorbital nerve injury, most clinicians depend on the patient’s subjective symptoms or judgements, lacking a generalized and objective evaluation method. Due to the limitations in subjective evaluations for accurate diagnosis of infraorbital nerve injury, we used the blink reflex to objectively evaluate injury to the infraorbital nerve. The causes of symptoms of ION injuries include soft tissue edema and nerve traction, contusion, or rupture [7]. Depending on the mechanism and extent of ION compression, individual fascicles within the ION can be variously affected. Thus, regions of sensory abnormalities may vary within the territory of the ION, eliciting ipsilateral paresthesia and numbness in the upper lips, perinasal area, and partial or entire cheek.

One of the most common causes of injury to trigeminal nerve branches, including the infraorbital nerve, is accidental or iatrogenic trauma. Approximately 30% to 80% of patients with midfacial fractures experience infraorbital nerve injuries [8]. Iatrogenic injury to the ION has also been reported in the setting of endoscopic sinus surgery, in which a canine fossa puncture for endoscope insertion is per-formed. In addition, the ION can be injured during a Caldwell–Luc procedure, when a facial flap is elevated in the area where the nerve exits the infraorbital foramen [9]. Previous studies have estimated that 60% to 80% of patients with maxillary fractures experience paresthesia immediately after trauma, and 15% to 30% have permanent sensory impairment [10,11]. One study reported that the incidence of paresthesia following ION injury was 38% to 85.7%, and that after surgery, it was 4.5% to 55%, with 14.8% of patients reporting that the paresthesia persisted for longer than 6 months [12]. Although the above cases of ION injury were mostly reported as secondary to trauma or surgical treatment, there have been no reports of isolated ION injury due to alloplastic facial implants. Midface augmentation with alloplastic implants is a common plastic surgery. In the United States, although approximately 8800 cheek implant procedures were performed in 2009, it would be challenging to accurately predict the incidence of hypoesthesia after implant surgery [13,14].

In general, radiological findings can confirm nerve compression or injury. However, if a lesion is not clearly observed in the radiological findings, it may not be easy for clinicians to determine whether surgery is needed to remove the implanted prosthesis. To evaluate nerve damage, sensory abnormalities are evaluated, in addition to the patient’s subjective symptoms. The current widely used methods to evaluate sensory abnormalities include two-point discrimination for sensory symptoms, static touch sensation, and thermal discrimination [15,16]. However, all of these tests have a disadvantage in that they are only measured according to the patient’s subjective judgment of the patient and are difficult to use as objective evaluation data for test results. Moreover, evaluation of sensation is not easy to perform in patients with soft tissue damage or facial edema; in noncooperative patients, it can be even more difficult to determine the degree of damage. Thus, in these situations, the blink reflex study is an important method for accurate diagnosis of nerve injury.

Blink reflexes are useful in detecting abnormalities, including not only proximal nerve segments for pathology types such as demyelinating or axonal neuropathies, but also central pathway lesions, including their central connections in the trigeminal and facial brainstem nuclei [17]. Therefore, the blink reflex study is conducted to determine the location of central nerve lesions (especially pontine or medullar) and peripheral nerve lesions (the trigeminal and facial nerves). Ohki et al. evaluated ION injury in maxillary lesions using the blink reflex study [5]. The latencies on both sides were not always abnormal, and they suggested that R1 is more effective than R2 in detecting lesions in the ION. In addition, patients with paresthesia were found to have an abnormal result in the blink reflex study. Other studies of ION injury following orbitozygomatic complex fractures showed an abnormal R1 response in 14 (70%) of 20 patients and abnormal R2 response in 9 (45%) patients [6]. In addition, Kang et al. conducted preoperative blink reflex studies on 16 patients complaining of sensory symptoms that were suggestive of ION injury, and 15 patients (93.7%) showed an abnormal R1, consistent with previous studies [18]. In the study by Kang et al., the blink reflex study was also performed in patients who had normal subjective symptoms before surgery, and they showed delayed latency in the test results of the blink reflex study [18]. A small sensory nerve injury may not be clinically detectable in patients with normal subjective symptoms but in abnormal findings on the blink reflex study [5].

To evaluate ION dysfunction, we applied the same method from Ohki’s study to stimulate the ION [5]. The patient in the present case displayed an abnormal blink reflex that manifested as ipsilateral delayed R1 and R2, indicating that the lesions along the ION pathway to the maxillary nerve impaired the afferent pathway of the blink reflex not only for larger fibers in the cutaneous nerve, but also for smaller fibers [5]. In the present case study, it is noteworthy that the ipsilateral R2 level from the postoperative blink reflex study was significantly different from the preoperative level. In general, the R1 fibers are related to tactile sensation, whereas the R2 fibers are involved with pain and temperature sensation [19]. The tactile sensation activates larger fibers in the cutaneous nerve of the Aβ fiber, while pain and temperature sensation activate the smaller fibers of the Aδ and C fibers [20,21]. Therefore, the thicker and larger the fiber is, the more susceptible it is to compression and inflammation. Fibers associated with R1 may be more vulnerable, so R1 fibers frequently demonstrate abnormal results on the blink reflex study. However, as in the present case, although the ION was intact around the infraorbital foramen, damage to the peripheral branch of the left ION could not be clearly confirmed in the radiologic findings. The preoperative blink reflex study revealed that the ipsilateral R2 was much more delayed than that of R1, but the postoperative blink reflex study showed that the latency of R2 was significantly shorter than on the previous test. This provides evidence that the smaller fibers of the Aδ and C fiber have a susceptibility to lesions, as in our patient.

## 4. Conclusions

This is the first report of isolated ION injury following midface augmentation with alloplastic facial implants. In addition, we have demonstrated the clinical utility of a numerical value provided by the blink reflex study to confirm damage to the afferent pathway of the ION. Therefore, we report the usefulness of the blink reflex study in diagnosing isolated infraorbital neuropathy as an evaluation tool, especially in cases in which the clinical neurosensory test is difficult or radiological image study cannot clearly diagnose nerve damage, as in the present case.

## Figures and Tables

**Table 1:** Values of the patient with the blink reflex at the infraorbital nerve.

**Infraorbital Nerve (Preoperation)**			
**Stimulation Side**	**Ipsilateral R1 (ms)**	**Ipsilateral R2 (ms)**	**Contralateral R2 (ms)**
**Left**	12.8	39.5	39.2
**Right**	11.0	33.8	34.1
**Infraorbital Nerve (Postoperation)**			
**Stimulation Side**	**Ipsilateral R1 (ms)**	**Ipsilateral R2 (ms)**	**Contralateral R2 (ms)**
**Left**	12.2	32.2	34.8
**Right**	11.9	32.7	33.5

Values are presented as number. R1 = early wave, R2 = late wave, ms = millisecond.

## Data Availability

Not applicable.

## References

[1] Seckel B.R. (1994). Facial Danger Zones: Avoiding Nerve Injury in Facial Plastic Surgery. Can. J. Plast. Surg..

[2] Magliacano A., Fiorenza S., Estraneo A., Trojano L. (2020). Eye blink rate increases as a function of cognitive load during an auditory oddball paradigm. Neurosci. Lett..

[3] Shahbakhti M., Beiramvand M., Rejer I., Augustyniak P., Broniec-Wojcik A., Wierzchon M., Marozas V. (2021). Simultaneous Eye Blink Characterization and Elimination from Low-Channel Prefrontal EEG Signals Enhances Driver Drowsiness Detection. IEEE J. Biomed. Health Inform..

[4] Ren P., Ma X., Lai W., Zhang M., Liu S., Wang Y., Li M., Ma D., Dong Y., He Y. (2019). Comparison of the Use of Blink Rate and Blink Rate Variability for Mental State Recognition. IEEE Trans. Neural Syst. Rehabil. Eng..

[5] Ohki M., Takeuchi N. (2002). Objective evaluation of infraorbital nerve involvement in maxillary lesions by means of the blink reflex. Arch. Otolaryngol.-Head Neck Surg..

[6] Park Y.S., Kim W.J. (2013). Correlation between infraorbital blink reflex study and clinical neurosensory test for diagnosing infraorbital nerve injury following orbitozygomatic complex fracture. J. Korean EMG Electrodiagn Med..

[7] Sakavicius D., Juodzbalys G., Kubilius R., Sabalys G.P. (2008). Investigation of infraorbital nerve injury following zygomaticomaxillary complex fractures. J. Oral Rehabil..

[8] Noor M., Ishaq Y., Anwar M.A. (2017). Frequency of infra-orbital nerve injury after a zygomaticomaxillary complex fracture and its functional recovery after open reduction and internal fixation. Int. Surg. J..

[9] Ference E.H., Smith S.S., Conley D., Chandra R.K. (2015). Surgical anatomy and variations of the infraorbital nerve. Laryngoscope.

[10] Jungell P., Lindqvist C. (1987). Paraesthesia of the infraorbital nerve following fracture of the zygomatic complex. Int. J. Oral Maxillofac. Surg..

[11] Schultze-Mosgau S., Erbe M., Rudolph D., Ott R., Neukam F.W. (1999). Prospective study on post-traumatic and postoperative sensory disturbances of the inferior alveolar nerve and infraorbital nerve in mandibular and midfacial fractures. J. Cranio-Maxillo-Facial Surg. Off. Publ. Eur. Assoc. Cranio-Maxillo-Facial Surg..

[12] Gierloff M., Seeck N.G., Springer I., Becker S., Kandzia C., Wiltfang J. (2012). Orbital floor reconstruction with resorbable polydioxanone implants. J. Craniofacial Surg..

[13] Raschke R., Hazani R., Yaremchuk M.J. (2013). Identifying a safe zone for midface augmentation using anatomic landmarks for the infraorbital foramen. Aesthetic Surg. J..

[14] Fogaca W.C., Fereirra M.C., Dellon A.L. (2004). Infraorbital nerve injury associated with zygoma fractures: Documentation with neurosensory testing. Plast Reconstr. Surg..

[15] Taicher S., Ardekian L., Samet N., Shoshani Y., Kaffe I. (1993). Recovery of the infraorbital nerve after zygomatic complex fractures: A preliminary study of different treatment methods. J. Oral Maxillofac. Surg..

[16] Yaremchuk M.J. (2003). Facial skeletal reconstruction using porous polyethylene implants. Plast. Reconstr. Surg..

[17] Bernard J.M., Pereon Y. (2005). Nerve stimulation for regional anesthesia of the face: Use of the blink reflex to confirm the localization of the trigeminal nerve. Anesth Analg..

[18] Kang D.I., Park S.W., Choi J.H. (2010). Usefulness of the blink reflex study as a preoperative evaluation in the orbitozygomatic complex fracture. J. Korean Soc. Plast Reconstr. Surg..

[19] Kimura J., Aminoff M.J. (1999). The blink reflex as a clinical test. Electrodiagnosis in Clinical Neurology.

[20] Beise R.D., Kohllöffel L.U.E., Claus D. (1999). Blink reflex induced by controlled, ballistic mechanical impacts. Muscle Nerve.

[21] Ellrich J. (2000). Brain stem reflexes: Probing human trigeminal nociception. News Physiol. Sci..

